# Comparison of online, offline, and hybrid hypotheses of motor sequence learning using a quantitative model that incorporate reactive inhibition

**DOI:** 10.1038/s41598-024-52726-9

**Published:** 2024-02-26

**Authors:** Mohan W. Gupta, Timothy C. Rickard

**Affiliations:** Department of Psychology , University of California, La Jolla, San Diego, CA 92093-0109 USA

**Keywords:** Psychology, Human behaviour

## Abstract

Two hypotheses have been advanced for when motor sequence learning occurs: offline between bouts of practice or online concurrently with practice. A third possibility is that learning occurs both online and offline. A complication for differentiating between those hypotheses is a process known as reactive inhibition, whereby performance worsens over consecutively executed sequences, but dissipates during breaks. We advance a new quantitative modeling framework that incorporates reactive inhibition and in which the three learning accounts can be implemented. Our results show that reactive inhibition plays a far larger role in performance than is appreciated in the literature. Across four groups of participants in which break times and correct sequences per trial were varied, the best overall fits were provided by a hybrid model. The version of the offline model that does not account for reactive inhibition, which is widely assumed in the literature, had the worst fits. We discuss implications for extant hypotheses and directions for future research.

## Introduction

Multiple researchers have advanced the hypothesis that motor learning occurs *offline,* both during sleep^1–3^ and in more recent work during brief waking breaks^4–7^. Conversely, motor sequence learning has been posited to take place concurrently with task performance (i.e., *online* learning)^8,9^. Discrimination among those and related hypotheses should have fundamental implications for the properties of the underlying neural system.

The hypothesized motor learning during waking breaks is believed to involve facilitating *micro-consolidation*, as opposed to the stabilizing consolidation (i.e., protection from forgetting and memory integration) that is thought to occur for declarative learning. The conclusion favoring that hypothesis is based on the findings that (1) there is often negative or no response time improvement over motor sequence repetitions within a performance trial, (2) performance at the beginning of a trial is often better than that at the end of the preceding trial, and (3) neural evidence of hippocampal replay appears to occur during rest periods.

The micro-consolidation hypothesis was challenged, however, by Gupta and Rickard^8^. They advanced evidence for a diametrically opposed learning model that assumes (1) that all learning occurs online, concurrently with task performance, and (2) that reactive inhibition (RI) accrues over sequences within a trial and dissipates over time during breaks. Although the mechanism underlying RI is not well established, the empirical effect it describes has been replicated over studies spanning more than 80 years^9–12^. Nevertheless, the phenomenon has not played a central role in recent studies of facilitating micro-consolidation. Gupta and Rickard^8^ explored the sufficiency of their hypothesis using a standard finger tapping task where participants repeatedly typed a five-key sequence with their non-dominant hand^13^. There were four groups, crossing 10 or 30 s per performance trial with a 10 or 30 s break between trials, while equating total time on task. After the training phase, there was a 5-min rest period, followed by additional trials. They found strong evidence during training for both accrual and dissipation of RI in all groups. Further, they observed that the largest RT gain (i.e., decrease in RT) in correct sequence completion time occurred in the 30 s on-task, 10 s break group and the smallest in the 10 s on-task, 30 s break group. These results are in-line with the hypothesis that RI builds-up substantially across training trials in the former group (because the 10 s breaks were insufficient to fully resolve the build-up of RI during each 30 s trial), but largely resolved during breaks in the latter group. Those results can account for the behavioral evidence underlying the micro-consolidation hypothesis without the need to posit offline learning.

Although Gupta and Rickard concluded that the online learning plus RI hypothesis may be sufficient to account for the ordinal pattern of post-rest RT gains in their data, they did not offer a quantitative model of the complex patterns that were observed across sequences, trials, and the rest period. Indeed, no such model has been advanced to date for an explicit motor sequence learning task (for a model of RI effects in the implicit motor sequence task, see Torok et al.^12^). If achievable with a modest number of psychologically plausible free parameters, a quantitative learning models should advance research in this area by (1) assessing the sufficiency of different models at a finer temporal grain size than in past work, (2) providing new insights into the properties of RI, and (3) setting a new reference for future theory development. Here we advance three types of learning models: online, offline, and hybrids of the two.

**Figure 1:**
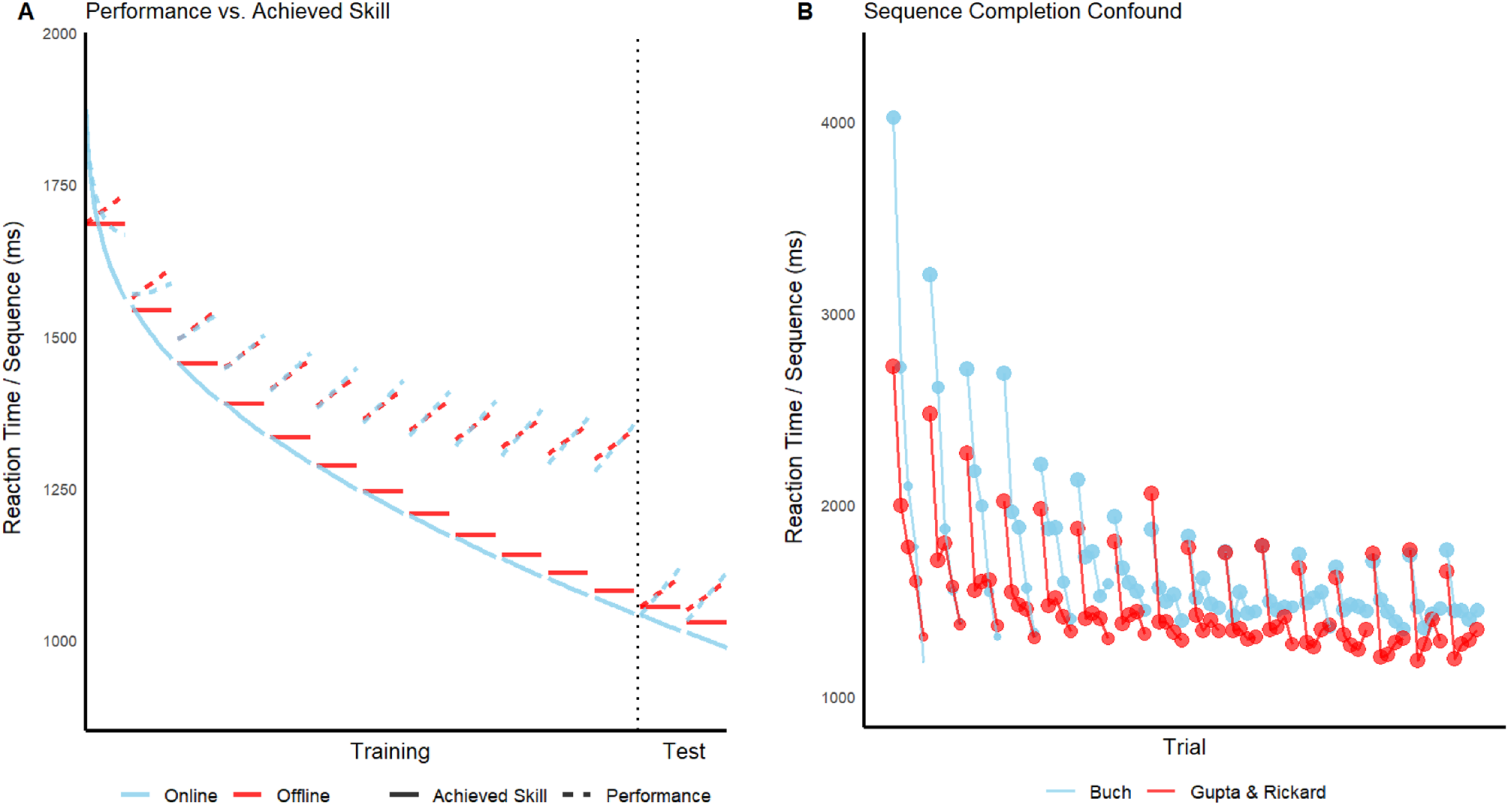
(**A**) Model predictions of the Online and Offline models assuming RI with respect to both underlying achieved skill (solid lines; Eqs. 1 and 6b) and observed performance as moderated by accrual and dissipation of RI (dashed lines; Eqs. 5 and 6c). (**B**) Data from Buch et al. (2019) and Gupta and Rickard (2022) are plotted for the first 16 trials. Both datasets consist of 10 s trials and 10 s breaks. Those data are plotted as a function of how quickly a sequence is completed, artificially truncated at five sequences per trial. Many participants completed more than five sequences. Because the constraint on all of these trials is time rather than number of correct sequences, participants complete a variable number of sequences, as evidenced by the size of the circles: the larger, the more participants that have completed that sequence number. Data of this type would unnecessarily complicate model fitting to averaged data, particularly over the first 11 trials in spaced conditions, which much of the prior work on the micro-consolidation hypothesis has been focused.

The motor sequence task used here is closely related to that used by Gupta and Rickard and by many prior authors. As one exception, a trial in the current study was defined as a fixed number of correctly completed sequences, rather than a fixed amount of training time. This change was made because in the time constrained version of the task, participants complete a variable number of correct sequences during each trial, complicating model fitting to averaged data (Fig. 1B). In the two *spaced practice* (S) groups each trial ended after completion of 5 correct sequences and in the two *massed practice* (M) groups, each trial ended after the completion of 15 correct sequences. We indicate break time between trials, in seconds, as the number after either M or S; hence, the group labels are S30, S10, M30, and M10. The total number of correct sequences (henceforth, *sequences*) was held constant across groups by implementing three trials in the spaced groups for every one trial in the massed groups, for a total of 180 training sequences and 30 test sequences in each group. Training and test phases were separated by a five-minute distractor task of double digit addition.

### Quantitative models

We first develop the general quantitative framework in the context of the Online model and then develop the Offline and Hybrid learning models. All models that include RI have an identical quantitative implementation. All models feature a variant of a *skill* function for mapping learning onto correct sequence completion time (henceforth, response time, or *RT*). Prior work on skill learning indicates that, for tasks that do not exhibit major strategy shifts with practice^14^, RT gain over trials is a smooth, negatively accelerating function of repetition. We adopted that assumption here. Because the exact mathematical function that governs achieved skill is unknown for motor sequence learning, we adopted a flexible function that combines power and exponential terms (see Supp 3; also advanced as a general practice function for cognitive skills^15^). Depending on the model, achieved skill occurs as a function of sequence practice (online learning), micro-consolidation during breaks (offline learning), or both (hybrid learning).

In all models we assume that no forgetting occurs during either the short breaks between trials or the 5-min rest period prior to the test. We further assume that the five minute post-training rest is sufficient to resolve all residual RI built-up across training trials. In the hypothetical case of no RI, the observed RT would directly reflect the achieved skill. However, in the presence of RI, achieved skill is a latent variable on all trials except on the first training trial and the first test trial, as elaborated below.

### Online skill model

The Online learning model assumes that learning starts immediately on the first sequence of a trial and runs to completion by the last sequence, such that there is no learning during breaks. In this model, RT for the underlying skill is solely a function of cumulative sequences over training and test phases (S),

1 $${\text{RT}}_{{{\text{skill}} - {\text{Online}}}} = a + b*{\text{e}}^{{( - c*{\text{S}})}} *{\text{S}}^{ - k} .$$

As motivated later, we removed the first sequence of the first trial (and of later trials) as a warm-up sequence and then started the cumulative sequence variable S at a value of one on the second sequence of the first trial; i.e., modeled sequence number one was actually experimental sequence two. Because there was a sequence learning event prior to modeled sequence one, Eq. (1) is parameterized appropriately. Parameter *a* is the asymptotically achievable skill, *b* is the improvement that can occur with practice, *c* is the exponential rate parameter, and *k* is the power rate parameter (for a summary of the estimated parameters and known variables across all models, see Table 1). Together, the two rate parameters determine the shape of the skill curve and how quickly it approaches asymptote. In the absence of RI, Eq. (1) would describe the *observed* RTs. That skill curve is illustrated by the blue solid line in Fig. 1A.

**Table 1:** Parameter and variable descriptions in the model equation. All parameters are constrained to have values > 0; j < Stot/Btot.

		Description
Parameters	*a*	asymptotic skill
	*b*	magnitude of skill improvement that can occur with practice
	*c*	Power rate
	*k*	Exponential rate
	*y*	rate of the RT slowing effect over sequences within each training and test trial due to the build-up of within-trial RI
	*z*	rate of the RT slowing effect over training and test trials due to build-up of residual RI
	*g*	additional increment in offline learning during the post-training rest period in version 2 of the offline model
	*j*	index of offline learning in the HybridJ model
Variables	S	modeled sequence number, cumulative across training and test phases
	ST	modeled sequence number within trial (1–14 for massed groups; 1–4 for spaced groups)
	T	training phase trial number (1 to N)
	T_test_	test phase trial number, starting a 1 on the first test trial
	X	dummy variable that takes a value of 0 for training trials and 1 for test trials
	B_tot_	total number of breaks including the rest period
	S_tot_	total number of modeled sequences
	S’	describes the allocation of learning units between online and offline components in the HybridJ model

#### Modeling RI

RI was implemented in the same way for all models. Two effects on the observed RT were implemented equivalently across all models that incorporate RI. The first is the increase in RT over sequences within each trial due to the build-up of RI (i.e., the *within-trial* RI effect). We assumed the simplest case of linear RT increase over sequences within-trial, with the same rate parameter (*y*) for all trials across both training and test phases. Although this linear effect may not hold when there are a very large number of sequences per trial, prior work^8,9^ suggests that it is a reasonable approximation for the current experiment. We assume that RI operates with the same force and magnitude across all trials.

The second effect is the build-up of residual RI from trial to trial due to its incomplete dissipation during trials (parameter *z*). Consider the M10 group, in which there are 15 experimental sequences within each trial and 10 s breaks between trials. Based on prior work, that group should exhibit both substantial within-trial RI build-up and incomplete resolution of RI during the short break, yielding a residual RI effect at the beginning of the next trial. That residual RI is expected to accrue over trials, and for simplicity we assume that the accrual rate is constant across trials. Thus, the difference between the observed RT and RT_skill_ on the first modeled sequence of each trial increases as a linear function of trial number during both training and test phases. That residual build-up should be largest for the M10 group and smallest for the S30 group, as implied by the results of Gupta and Rickard^8^. Given a linear relation between RI and its effect on RT, the predicted RT change within and across training trials due to RI is,

2 $${\text{RT}}_{RI - training} = \left( {{\text{ST}} - {1}} \right)*y + \left( {{\text{T}} - {1}} \right)*z,$$

where (ST-1) reflects the fact that within-trial RI is defined to be zero on the first sequence of each trial, and (T-1) reflects the fact that residual RI is by definition zero on trial one. The effect of within-trial RI on RT is illustrated in Fig. 1A, where the effect of the residual RI build-up is illustrated by the widening gap over trials between the dashed and solid curves.

Based on our earlier work^8^, we assume that a 5-min rest period is sufficient to completely resolve residual RI for all four groups. Hence, the observed RT for the first test phase sequence is expected—like that on the first training trial—to be a pure measure of RT_skill_ (see Fig. 1A). The build-up of both within-trial and residual RI during the test, and the consequential RT effects, are assumed to occur at the same rate as during training. Thus, for the test phase, the effect of RI on RT is:

3 $${\text{RT}}_{{{\text{RI}} - {\text{test}}}} = \left( {{\text{ST}}{-}{1}} \right)*y + \left( {{\text{T}}_{{{\text{test}}}} {-}{1}} \right)*z,$$

where T_test_ is the test trial number, which starts at a value of 1.

Hence, the effect of RI across both training and test phases is given by the mixture equation:

4 $$RT_{RI} = \left( {{1} - {\text{X}}} \right)*\left\{ {\left( {{\text{ST}} - {1}} \right)*y + \left( {{\text{T}} - {1}} \right)*z} \right\} + {\text{X}}*\left\{ {\left( {{\text{ST}} - {1}} \right)*y + \, \left( {{\text{T}}_{{{\text{test}}}} - {1}} \right)*z} \right\},$$

where X takes a value of zero during training and one during the test.

#### Full online model equation

The simultaneous least squares nonlinear model fitting across both training and test phases was accomplished separately for each model and group by combining the appropriate skill equation for the model with the common RT_*RI*_ equation. For the Online model:

5 $${\text{RT}}_{{{\text{overall}} - {\text{Online}}}} = {\text{RT}}_{{{\text{skill}} - {\text{Online}}}} + {\text{RT}}_{{{\text{RI}}}}$$

### Offline model

This model assumes that learning occurs exclusively during breaks and that the amount of learning is equivalent across all of the equal duration breaks. The proposed micro-consolidation account^5–7,16^ is consistent with both assumptions, given that hippocampal replay occurs at the same rate across all breaks. In this model, S in the online skill equation is replaced by T-1, given that learning occurs during breaks between trials,

6a $${\text{RT}}_{{{\text{skill}} - {\text{Offline}}}} = a + b*{\text{e}}^{{\{ - c*({\text{T}} - {1})\} }} * \left( {{\text{T}} - {1}} \right)^{ - k} ,$$

where (T-1) places the first offline consolidation event appropriately after the first trial.

Two versions of the Offline model were under primary consideration. First, because the reference Offline model in the literature assumes negligible effects of RI on performance, we considered a version (V1) with no RI. The overall equation for observed RT in this case is just the offline skill equation (Eq. 6a):

6b $${\text{RT}}_{{{\text{observed}} - {\text{Offline}} - {\text{V1}}}} = {\text{RT}}_{{{\text{skill}} - {\text{Offline}}}}$$

In version 2 we added RI to version 1:

6c $${\text{RT}}_{{{\text{observed}} - {\text{Offline}} - {\text{V2}}}} = {\text{RT}}_{{{\text{skill}} - {\text{Offline}}}} + {\text{RT}}_{{{\text{RI}}}} .$$

We also considered variants of those two offline models in which more micro-consolidation occurs during the 5 min rest period than during the training phase breaks. Given that X (see Table 2) takes a value of zero during training and of one during the test, and representing the RT effect of extra consolidation during the rest period with a offset new parameter (*g*), the skill equation for that variant is,

6d $${\text{RT}}_{{{\text{skill}} - {\text{Offline}} - {\text{offset}}}} = a + b*{\text{e}}^{{\{ - c*({\text{T}} - {1})\} }} *\left( {{\text{T}} - {1}} \right)^{ - k} - g*{\text{X}}$$

**Table 2:** For each model the BIC was calculated. Models with better fits, penalizing for the number of parameters have lower BIC values. Numbers in bold indicate the best fit for that group, whereas italicized indicates worse fits for that group.

Group	Offline No RI	Offline	Online	HybridJ	HybridE	HybridP
S30	*1315.558*	**1229.712**	1241.242	1232.569	1240.359	1254.786
S10	*1477.377*	1294.249	**1279.425**	1284.630	1279.534	1293.744
M30	*1545.205*	1475.403	1501.229	**1468.726**	1484.094	1472.465
M10	*1611.154*	1597.050	1569.606	**1569.044**	1569.079	1595.570
Mean	*1487.323*	1399.103	1397.876	**1388.742**	1393.266	1404.141

When that variant was fitted in the absence of assumed RI, in no group was the fit better by the Bayesian Information Criterion (BIC) than that for offline model version 2. When that variant was fitted with RI included, the residual sum of squares (RSS) was slightly reduced relative to offline model version 2, but version 2 again provided better BIC fits. We thus do not consider those two variants further in this paper.

### Hybrid models

A third class of models assumes that learning can occur both online and offline. This assumption is plausible because multiple systems are likely to underlie motor sequence learning^17–19^. Three variants are considered: *HybridJ, HybridE, and HybridP*.

#### HybridJ

The goal of the HybridJ model is to estimate the relative proportion of learning that is due to offline and online components. Conceptually, each executed sequence across both training and test phases is treated as yielding one unit of learning. If both offline and online learning occur, then some of those learning units occur concurrently with sequence execution and some occur during the break periods. As is implicitly the case for the Online and Offline models, the number of learning units per trial or break is held constant over practice and test phases, such that the non-linear RT improvement (skill) is solely a property of the mapping from learning to performance. It may be more accurate to assume that the learning rate itself decreases across practice sequences and trials. However, we cannot differentiate between those two possibilities in the current work. For convenience, we implement the non-linear effect fully within the RT skill equation and assume that the underlying learning increments are constant over all sequences and trials.

A single new parameter (*j*) estimates the number of sequence learning units that occur during each break (offline learning). Defining B_tot_ as that total number of breaks in each group (including the 5-min rest period), then B_tot_*j is the total number of (offline) learning units that occur during all breaks. In the massed groups, for example, there are 11 breaks during training, a break between training and test phase, and a break between the first and second test trials, so that B_tot_ = 13. The number of remaining sequence-level learning units available for online learning is then S_tot_–B_tot_*j, where S_tot_ is the total number of modeled sequences across training and the test phases.

The cumulative effective number of learning units accrued across sequences and trials is then,

7a $${\text{S} ^\prime} = {\text{S}}*\left( {{\text{S}}_{{{\text{tot}}}} {-}{\text{B}}_{{{\text{tot}}}} *j} \right)/{\text{S}}_{{{\text{tot}}}} + \left( {{\text{T}} - {1}} \right)*j,$$

where (T-1)*j implements the cumulative number of sequence learning units that occur across breaks (offline) and S*(S_tot_–B_tot_*j)/S_tot_ implements the remaining fractional learning units that accrue across sequences. The total number of sequence units is conserved, so that on the last test sequence, the value of S’ converges on the value of S. In this HybridJ model, S’ replaces S in the skill equation, yielding,

7b $${\text{RT}}_{{{\text{skill}} - {\text{HybridJ}}}} = a + b*{\text{e}}^{{( - c*{\text{S}}^\prime)}} *{\text{S}}{^\prime}^{ - k} ,$$

and the observed RT equation is,

7c $${\text{RT}}_{{{\text{overall}} - {\text{HybridJ}}}} = {\text{RT}}_{{{\text{skill}} - {\text{HybridJ}}}} + {\text{ RT}}_{{{\text{RI}}}} .$$

If all learning is either online or offline, then we expect the HybridJ model to yield a higher (less favorable) BIC value than either for the Online or Offline model, due to the extra free parameter, *j,* which in those cases would not yield improved fits. Conversely, if both types of learning play an important role, then we expect a best fit of this hybrid model with the estimated value of *j* somewhere between zero (all online learning) and its maximum value of Stot/Btot (all offline learning). HybridJ is also appropriate for a variant of the Online model not considered earlier, in which all learning is triggered by the act of performance but in which learning runs to completion over time that includes break periods.

#### HybridE and HybridP

The HybridE and HybridP models assume that there is both offline and online learning, but they posit that those two terms of the skill equation map exclusively to either online or offline learning. Hence, these two models and the HybridJ model address independent rather than competitive hypotheses. In the HybridE model, the exponential RT improvement [e^(-c*T-1)^] occurs offline and power RT improvement [S^k^] occurs online.

8a $${\text{RT}}_{{{\text{skill}} - {\text{HybridE}}}} = a + b*{\text{e}}^{{\{ - c*({\text{T}} - {1})\} }} *{\text{S}}^{ - k} ,$$

yielding,

8b $${\text{RT}}_{{{\text{overall}} - {\text{HybridE}}}} = {\text{ RT}}_{{{\text{skill}} - {\text{HybridE}}}} + {\text{ RT}}_{{{\text{RI}}}} .$$

The HybridP model assume the reverse,

8c $${\text{RT}}_{{{\text{skill}} - {\text{HybridP}}}} = a + b*{\text{e}}^{{( - c*{\text{S}})}} *\left( {{\text{T}} - {1}} \right)^{ - k} .$$

yielding,

8d $${\text{RT}}_{{{\text{overall}} - {\text{HybridP}}}} = {\text{RT}}_{{{\text{skill}} - {\text{HybridP}}}} + {\text{RT}}_{{{\text{RI}}}} .$$

## Results

### Errors

**Figure 2:**
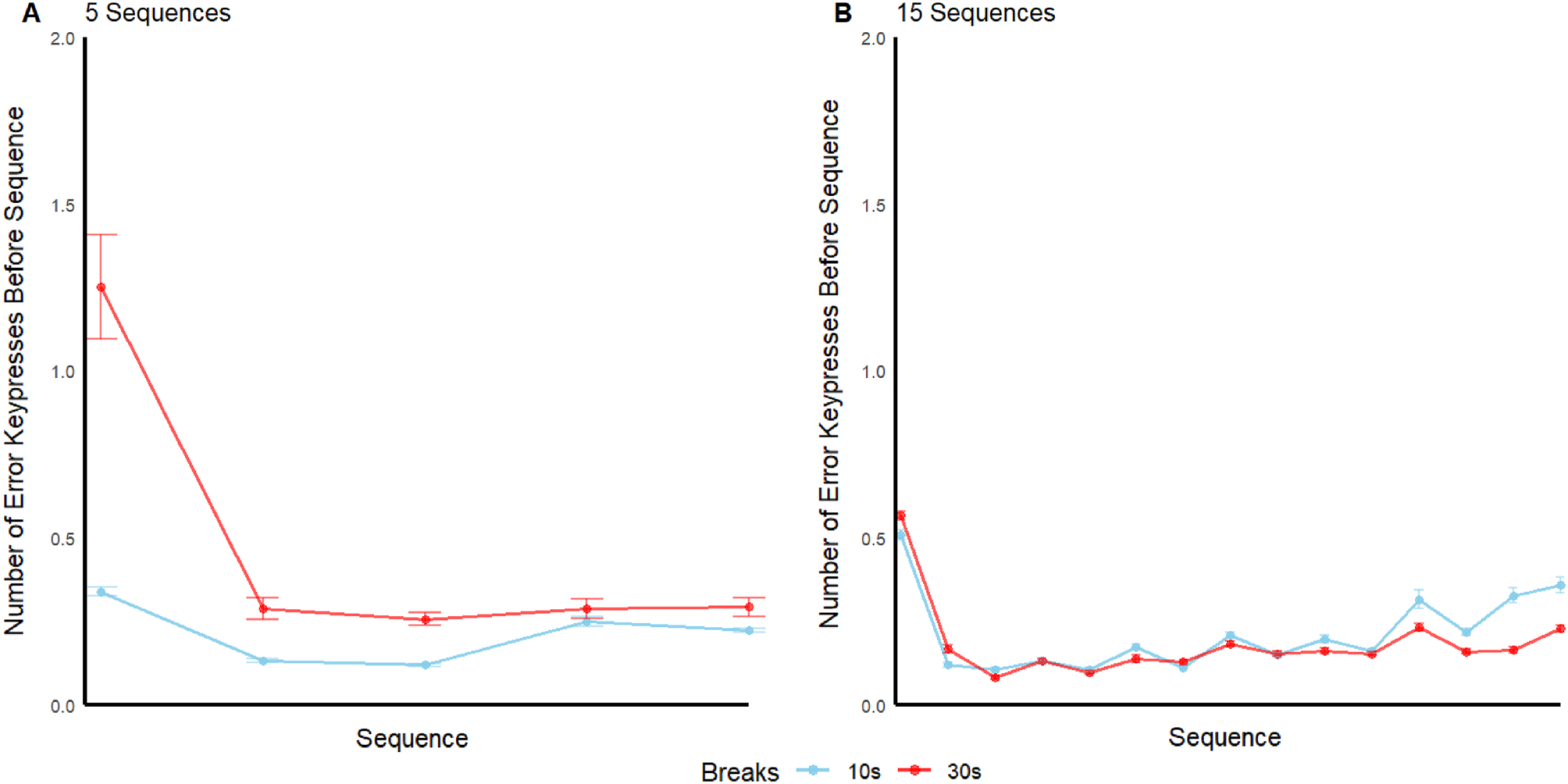
The x-axis corresponds to the number of the sequences completed within the trial. The y-axis is the average number of error keypresses before a completed sequence. For example, the first data point refers to the average number of error keypresses before the first sequence. Error bars are standard error.

The error rate was calculated for each participant as the number of incorrect key presses prior to each (correct) sequence within each trial. Averaging over sequences, trials, and participants in the training phase, the error rate was 0.17, 0.20, 0.39, and 0.20 key presses in the M30, M10, S30, and S10 groups, respectively. Given five key presses for a correct sequence, the proportional key press error rate is ranged from 0.033 to 0.073 across groups. A mixed factors Factorial Analysis of Variance (ANOVA) on the error rate revealed no effect of either trial type (massed vs. spaced), F(1, 164) = 0.71, p = 0.4, d = 0.0043 or break time (10 s vs. 30 s), F(1, 164) = 0.3, p = 0.59, d = 0.0018, and no interaction F(1, 164) = 0.71, p = 0.4, d = 0.0043. The mean error rates across sequences within-trial, averaged over training trials and participants, are depicted in Fig. 2. The Error rate prior to the first sequence was relatively high for all groups, suggesting a “warm-up effect” on performance at the beginning of each trial. For the 5 sequence groups, the number of errors made prior to sequences 2 through 5 is roughly constant with no significant Pearson correlation between error rate and sequence number, r(330) = 0.032, p = 0.56. However, for the 15 sequence groups, there is a gradual increase in error rate from sequence 2 onward, r(1188) = 0.12, p < 0.0001. This suggests that in the 15 sequence groups only, within-trial RI manifested not just in correct sequence RTs but also to some extent in the error rate.

### Correct sequence RTs and model fits

**Figure 3:**
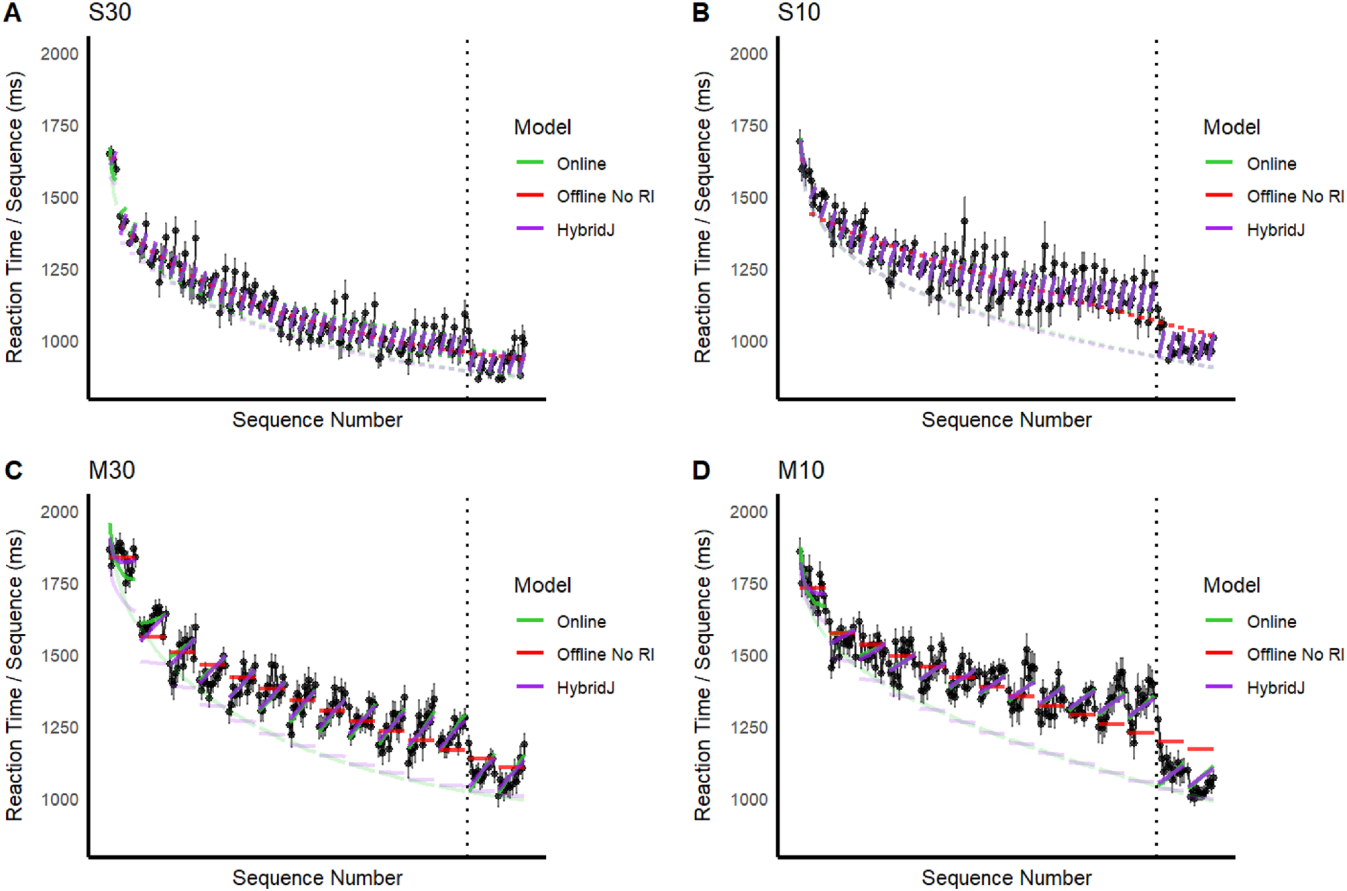
Each blackdot is the RT of one completed sequence. Error bars are the standard error. The darker lines depict the overall model fits. The faint lines underneath are the estimated achieved skill.

As shown in Supplement 1, the first sequence of each training and test trial had RTs that were far longer than that for other sequences, mirroring the higher error rate prior to those sequences. Those outlier sequences were removed prior to model fitting. Individual participant RTs can be seen in Supp 8. The mean sequence RTs over participants are shown in Fig. 3 for all groups, along with fits of the Online model, Offline model version 1 that assumes no RI, and the HybridJ model. Version 1 of the Offline model is unable to capture either the pronounced RI effects (RT increase) over sequences within-trial or the prominent residual RI effect across trials, particularly for the massed groups. However, the Online and HybridJ models were also able to capture those and other major patterns in the data. Note the curvilinear RT prediction over sequences for the first few trials that is most prominent for the Online model and present for the HybridJ model but is absent for the Offline model version 1 (as well as version 2). That curved form reflects the combined effects of the linear RI effects over sequences and the non-linear decrease on RT skill curve. For all of the model fits overlaid, see Supplement 5.

Also evident in Fig. 3 are at least two deviations of the data from the predictions, even for the best fitting HybridJ model (see discussion below), that seem unlikely to reflect chance. First, RTs on the first test trial are systematically larger than the model predictions. This suggests that there is a longer warm-up period after a 5-min rest than after 10 or 30 s breaks between trials during training (there was no warmup trial prior to the test). Second, for the M10 group, the predicted RTs on the last test trial are longer than the observed RTs for all sequences.

Model fits to correct sequence RTs were assessed using the Bayesian Information Criterion (BIC) applied to least-squares parameter estimation,

$${\text{BIC }} = {\text{ n}} * {\text{ln}}\left( {{\text{RSS}}/{\text{n}}} \right) + {\text{h}} * {\text{ln}}\left( {\text{n}} \right),$$

where lower values correspond to better fits. This criterion combines evidence of fit quality (the residual sums of squares; RSS) with a penalty for model variants with more free parameters (h). Results of each group and model, along with the mean BIC scores over groups for each model, are shown Table 2.

Version 1 of the Offline model, which assumes no RT and best represents the micro-consolidation account in the literature, yielded the worst fits across all models for all four experimental groups. In the rankings described next, we will ignore that model. Version 2 of the Offline model that assumed RI provided the best fit to the S30 group, the worst fit to the S10 group, an intermediate fit to the M30 group, and the second worst fit to the M10 group. The Online model yielded better fits overall than the Offline model. It provided the best fit for the S10 group, intermediate fits across models for the S30 and M10 groups, and the second worst fit for the M30 group.

Those results are qualified, however, by those for the hybrid models. HybridJ provides the best fits overall, including the best fits for the M10 and M30 groups. In those fits parameter *j* provides an estimate of the proportion of the observed RT improvement that is due to the Offline model (version 2) as opposed to the Online model. The fitted values of *j* were 4.097, zero, 13.42, and 11.81 for the S30, S10, M30, and M10 groups, respectively (Supp 4). Hence, the HybridJ fits suggest that 100%, 0%, 89%, and 78% of the learning, respectively for those groups, was due to offline learning. Those results suggest a larger contribution of offline learning for three of the four groups, and fully Online learning for the S10 group. We take those results as preliminary, given the relatively low correlation coefficients of the j parameter in our parameter recovery analysis (Supp 7).Fine grained estimation of the relative influence of online and offline components awaiting further research.

Given that the HybridJ results suggest both online and offline learning, the HybridE and HybridP models can address the complementary question of whether the power and exponential components of the skill function might selectively map onto those two types of learning. The substantially better BIC fits for HybridE compared to HybridP, combined with the second-best fits overall for HybridE, suggests tentatively that online learning may manifest mostly as power RT gains, whereas offline learning may manifest mostly as exponential RT gains. We will return to that finding in the Discussion.

### Reactive inhibition and the skill function across groups

For simplicity in characterizing the patterns for RI, we will focus here on the Online model. The following patterns held across all models that included RT with minor variations in the estimated parameter values (Supp 4). First, in the context of both massed and spaced training, the rate of within-trial RI build-up, as estimated by the parameter *y*, was minimally related to break time. This results suggests that the within-trial RI effect is relatively independent of the rate of accrual of residual RI over trials, which was greater in the massed groups. In contrast, the *y* estimates were significantly smaller for the two massed practice (M) groups (11.68 and 8.11) than for the two spaced practice (S) groups (28.52 and 32.25). Hence, the rate of per sequence RT increase was substantially larger than in the spaced groups, even though total slowing over sequences and accrual of residual RI was greater in the massed groups. That result was not expected. However, in light of the increasing error rate over sequences for the massed but not the spaced groups (Fig. 2), it may reflect a speed-accuracy trade-off for the massed groups. In that speculative account, participants in massed groups achieved a smaller rate of RT increase across sequences within-trial at the expense of a progressively increasing error rate over those sequences. The accrual of residual RI across trials (parameter *z*), differed across groups in an ordinal pattern that is consistent with expectations based on our prior work, having estimated values of 1.032 ms, 4.286 ms, 10.88 ms, and 19.12 ms per trial in the S30, S10, M30, and M10 groups, respectively.

**Figure 4:**
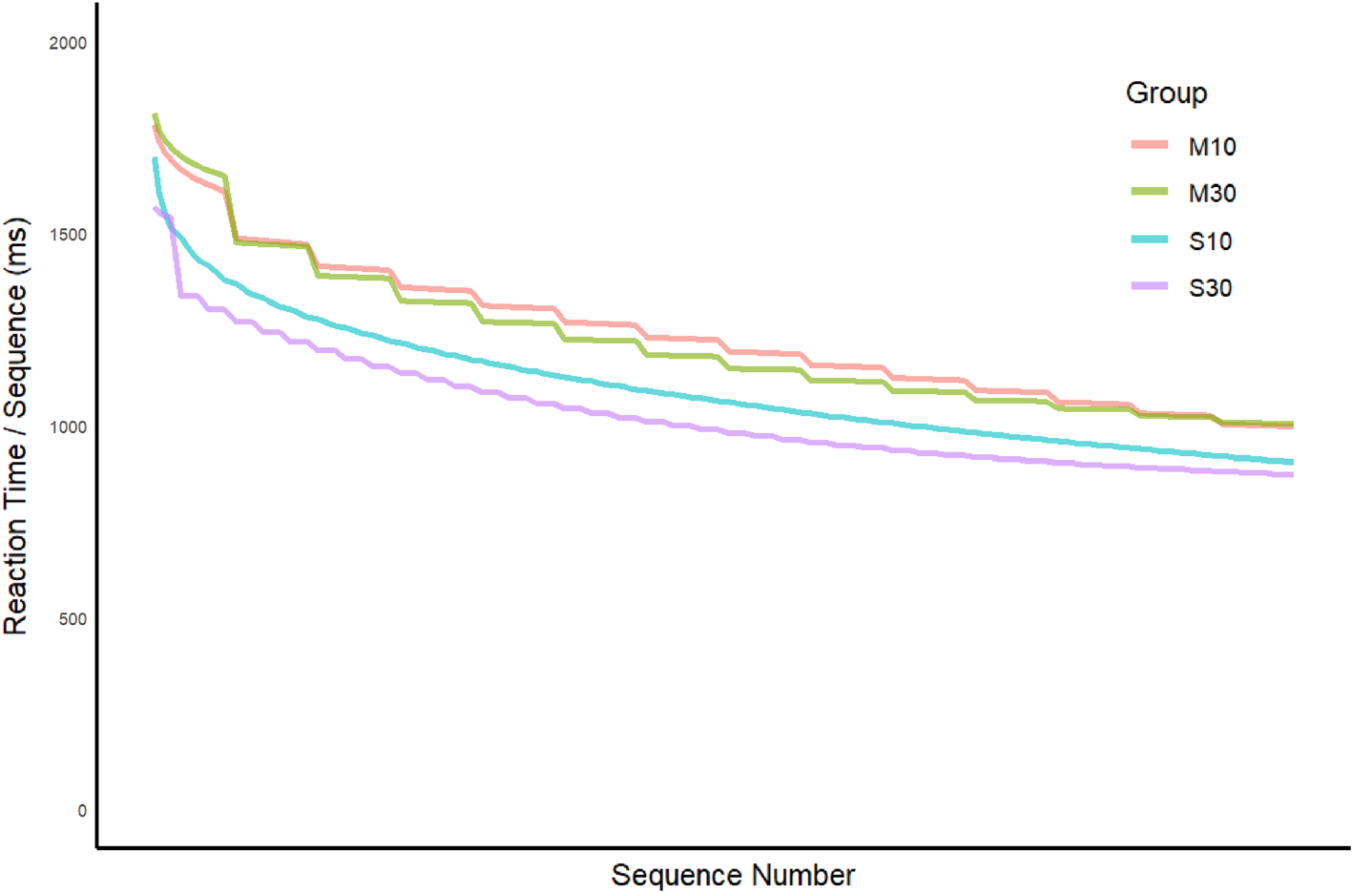
Achieved skill estimates from the HybridJ model for training trials for each group.

The results for achieved skill are best understood by plotting the curves for the four groups on the same graph (Fig. 4). In this comparison we used the HybridJ model fits as a reference, but similar patterns were observed for the other models (Supp 5). The curves are highly similar for the two massed groups, and similar, to a somewhat lesser degree, for the two spaced groups. In contrast, there is a clear gap between the massed and spaced groups throughout training. We will consider the theoretical implications of these results in the Discussion.

## Discussion

We explored three hypotheses for learning over the time course of explicit motor sequence practice. The models differ with respect to whether learning occurs online, offline, or both. Each model incorporates a flexible function for mapping learning to RT that includes both exponential and power rate parameters. Each involves the same quantitative treatment of both within-trial and residual RI (with the exception of the Offline version 1 model), with assumed constant magnitude within-trial and residual RI effects across training and test phases.

Comparison of the reference Offline models that did and did not account for RI confirmed that inclusion including RI yields much better fits. Across all models that did include RI, fit quality by the BIC values differed. The HybridJ model fitted best, suggesting both online and offline learning occur. Comparison of the HybridE and HybridP model fits favored HybridE, suggesting that online learning yields primarily power function RT gains, whereas offline learning yields primarily exponential RT gains.

### Reactive inhibition and skill learning

Although we expected the current finding that the residual RI over trials would be greatest in the M10 group and least in the S30 group, we were agnostic about the relative magnitude of that effect in the M30 vs S10 groups because the relative rate of RI accrual during performance vs. RI resolution during a break was not known. We observed greater residual RI build-up in the M30 group than the S10 group (see CIs for the z parameter in Supp 4), indicating that the difference in accrual of RI for the 15 vs. 5 sequences per trial exceeds the difference in the resolution of RI for a 10 s break vs. a 30 s break.

The clear difference in model fits between version 1 and version 2 of the Offline model strongly indicate the necessity of RI. Some authors have assumed or stated that RI is minimal on early training trials and becomes progressively more pronounced over trials once asymptotic performance has approached^5–7,9,16^. However, in the current models the linear effect of RI on RT within-trial was constrained to be the same across all training and test trials, yielding good overall fits. More generally, our results make it clear that modeling of RI effects should be central to any future work on the nature of motor sequence learning.

Given the relatively small step of RT improvement from the end of training to the beginning of the test for the S30 group, the estimated RT_skill_ curve for that group (Fig. 3) may be a close approximation of true underlying achieved skill, as anticipated by experimental design. Given that the achieved skill curves in that Figure are highly similar for the two spaced groups, we can tentatively conclude that the curve for the S10 group also approximates achieved skill on the first sequence of every trial. The longer RT_skill_ values in the massed groups in Fig. 4 is consistent with either of two interpretations, between which we cannot distinguish here. First, it may be that a 5-min break was not sufficient to fully resolve the accumulated RI for the massed groups, and that the residual RI that remained at the beginning of the test in those groups was absorbed into the achieved skill curve in the least squares fits. By that account, actual achieved skill may be the same in all four groups, but the “achieved skill” estimate for the massed practice groups is contaminated by persistent residual RI, yielding longer RTs. Alternatively, the curves for the massed groups may reflect a lower level of achieved skill; that is, it may be that the higher level of RI in the massed groups adversely affected not only the observed performance but also the amount of underlying skill learning. The question of whether RI affects both performance and learning has clear relevance for both learning theory and optimization of skill training, and warrants further investigation. A few early studies addressed that question for other types of motor skill tasks, but no strong consensus was reached^20–22^.

### Fast and slow learning

Our model includes two learning rate components, a power component that yields rapid RT gains during early practice and slower gains later and an exponential component that yields constant proportion RTs gains from sequence to sequence or trial to trial (i.e., relatively smaller RT gains early in practice; Supp 6). As noted earlier, those RT non-linearities are presumed in the current models to reflect solely the mapping from learning to RT. However, they may instead reflect differences between a *fast* learning process that yields early RT gains and a *slow* learning process that can yield more evenly distributed RT gains. The plausibility of separate fast and slow learning processes in motor sequence learning stems from the discovery of fast and slow processes in motor adaptation studies^17,18^. In those studies, the fast process has been linked to declarative (i.e., hippocampally mediated) learning and the slow process to nondeclarative (e.g., basal ganglia mediated) learning. In the HybridE model, we forced the fast power RT gains to occur exclusively online and the slower exponential gain to occur exclusively offline. In the HybridP model, we forced the reverse. Overall, the BIC results clearly favored the HybridE model between those two, and the overall fit of HybridE lagged only to the complementary HybridJ model. Those results suggest that in the motor sequence task, the fast declarative learning occurs online and the slower nondeclarative learning occurs offline. That conclusion, though speculative, appears to be inconsistent with a micro-consolidation perspective that assumes hippocampal to neocortical replay^7,16^–which presumably involves the same mechanism as does traditional declarative memory consolidation–occurs offline.

### Sleep research bearing on online vs. offline learning

There is a long-held belief that facilitating motor consolidation occurs during sleep^9,11,23–25^. However, when experimental design and analysis confounds–including RI, circadian rhythms, and averaging over online learning in the data analysis–are controlled for or mitigated, the post-sleep performance gain in motor sequence learning is virtually eliminated^9,11,23^. Most recently, meta-analytic evidence for substantial publication bias in that literature has been reported^25^. When the effects of both publication bias and confounding factors are simultaneously adjusted for, the data suggest that some degree of forgetting rather than performance improvement occurs during the hours-long offline periods of both wakefulness and sleep. The current results leave open the possibility of facilitating offline motor consolidation over brief waking periods but not over sleep periods.

### Limitations

The current study is subject to several limitations. First, it does not explain the slower than expected RTs across the first several sequences on the first test trial. We assume that is due to an extended “warm-up” effect. We also do not explain the mechanistic basis of the pronounced first sequence “warm-up” effect that was observed on all trials (Supp 1). Our approach to analysis in which those initial events of a trial are ignored is not unique in the literature, however, and it does not appear to compromise our main conclusions. Second, our inference that the lower rate of RI build-up over sequences within-trial in the massed groups may be a result of a speed-accuracy trade-off is speculative. Third, our use of an achieved skill function that includes two rate parameters is not strongly motivated a priori, although it is consistent with recent claims of separate learning rates for the declarative and nondeclarative components of motor adaptation learning^17,18,26,27^. As a practical matter, we used a function with two rate parameters because it yielded better data fits than did either the exponential or power function alone (Supp 3). Fourth, our assumption that the estimated achieved skill curve reflects the true latent skill level, while plausible for the spaced groups, is less certain for the massed groups, as discussed above. Fifth, although the HybridJ model had superior fits, in our model recovery analysis (Supp 7), the correlation between the recovered and known parameters was relatively low for the *j* parameter and others. Hence, one should approach the estimated values of j (reflecting both online and offline learning) with caution. Finally, we attempted to fit the non-linear models at the participant level, but were unable to achieve consistent convergence, and we further suspect that fits at that level are complicated by local minimum solutions. To assure optimal fits to the averaged data, we performed the non-linear fits independently in two statistical programming languages (R and SAS) – confirming that they both converged on the same RSS values—and in all fits we used an extensive starting parameter grid search prior to commencement of gradient descent.

### Directions for future work

Conclusive differentiation among the candidate models and estimation of the relative influence of online and offline learning will likely require experiments that are designed to specifically differentiate between parameter values and a shift to state-space modeling, along with datasets with decreased error variance. This will allow for greater ease of fitting a 7-parameter model, compared to our experiment which had little group differentiation.

State-space modeling would further elucidate how changes in learning and performance unfold over time. In the current context, there are at least three processes that may occur exclusively over time: dissipation of RI during breaks, saturation of offline learning during breaks, and saturation of online learning over time in the noted alternative online account in which learning is triggered immediately by performance but runs to completion during the break. In all three cases, initial modeling might assume a single-parameter exponential time function, with progression to more complex functions as justified by the BIC measure. The state-space approach will provide stronger constraints, and perhaps deeper biological insights, in subsequent work. Strategies for achieving more systematic data in future work include reducing the variability in participant-level sequence RTs (e.g., by increasing the number of key presses in each sequence), substantially increasing the participant sample size, and a closer examination of initial learning where predictions between the models are most varied (Supp 9) will be needed.

## Conclusion

Three classes of models based on when motor learning occurs were tested: online, offline, and hybrid. We showed the necessity of including RI as a central component in any such model. The results favor a hybrid model in which both online and offline learning occur. The quantitative modeling framework described here provides researchers with a new systematic and integrated approach to investigate mechanisms that underlie motor sequence learning and performance.

## Methods

### Participants

All 148 participants were right-handed. 42 participants in 15 correct sequences per trial, 30 s breaks (age = 20.38, F = 90.5%). 42 participants were in the 5 correct sequences per trial, 30 s break group (age = 20.83, F = 83.3%). 43 participants were in the 15 correct sequence per trial on, 10 s break group (age = 21.28, F = 79.1%). 41 participants were in the 5 correct sequences per trial on, 10 s break group (age = 20.02, F = 82.9%). Participants provided informed consent via button press. All procedures were approved by the institutional review board of the University of California, San Diego and all methods were performed in accordance with the relevant guidelines and regulations.

### Experimental design and procedure

**Figure 5:**
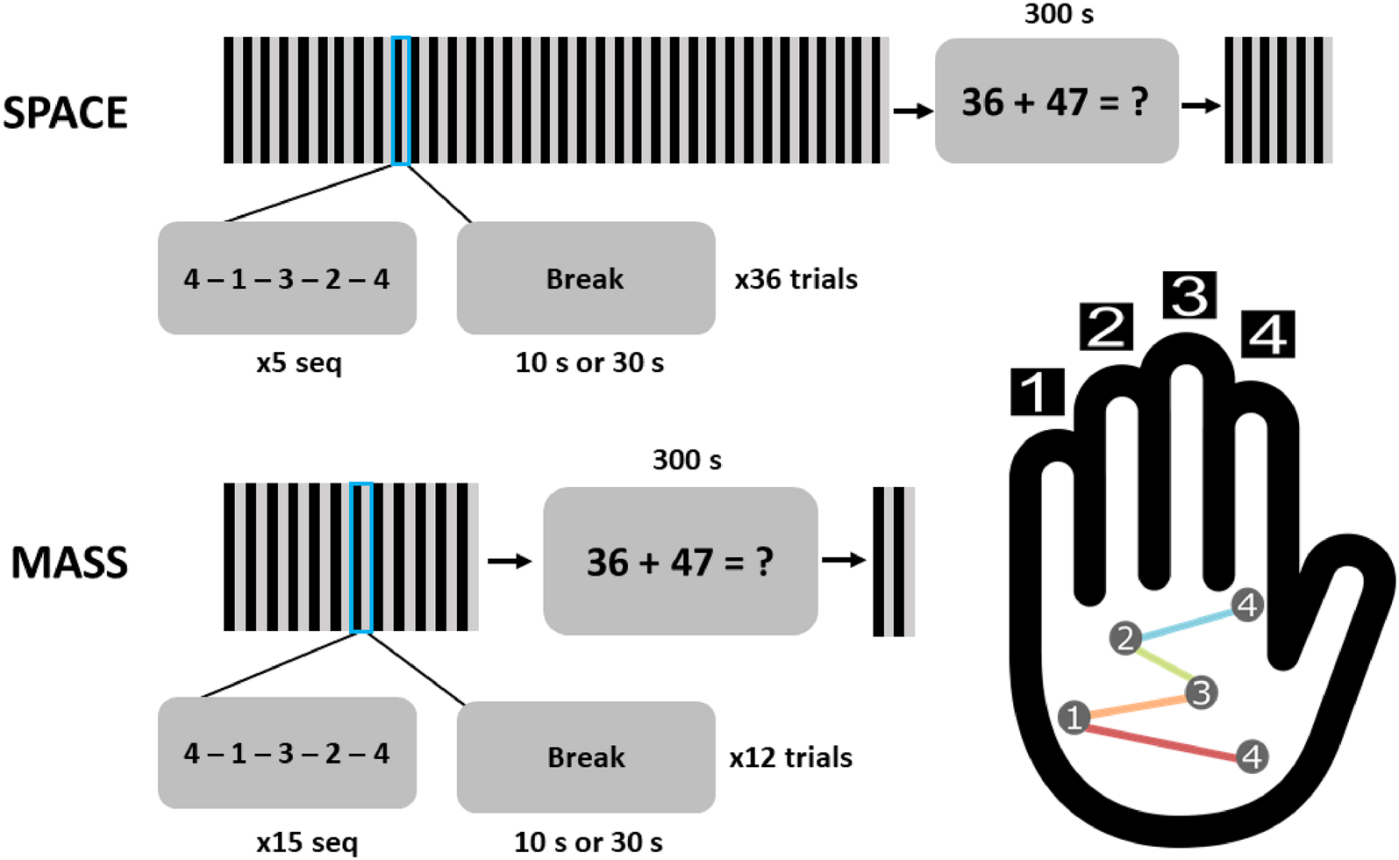
Finger tapping task. Participants learned a motor sequence task during a single session. They were instructed to repeatedly type a sequence, 41,324, with their non-dominant left hand as fast and as accurately as possible. Keypress 4 was performed with the index finger, keypress 3 with the middle finger, keypress 2 with the ring finger, and keypress 1 with the pinky finger. Participants trained for a total of 180 sequences with either 5 or 15 sequences per trial. In between practice trials were either breaks of 10 s or 30 s. After training, participants performed 300 s of double-digit addition. They were then tested on the practiced sequence for another 30 sequences with the same trial and break lengths during training.

Participants performed a standard finger-tapping-task where they repeated the sequence, 4-1-3-2-4 (see Fig. 5), as quickly and accurately as possible with their non-dominant left hand^13^. A 2 × 2, between-participant design was used, with factors of Number of Sequences (5 or 15 sequences) and Break Period between trials (10 s or 30 s). All participants completed the same total number of sequences. After the 180 completed sequences of on-task training, there was a 300 s rest where participants performed a distraction task of double-digit addition. Afterwards, they performed 30 sequences of test trials with breaks in between in the same conditions that they trained on. With this design, we were able to replicate our previous results that used a time based constraint (Supp 2; Gupta and Rickard^8^).

### Statistical analysis

The first completed sequence of each trial was considered a warm-up sequence and was removed prior to the RT data analysis^8^ (see fig S2). Thus, participants in the 15 sequence group had more data analyzed because they had fewer trials.

Keypress latency was defined as the time between temporally adjacent keypresses. We first log-transformed individual keypresses for correct sequences. The log-transformation reduced noise without changing the overall data pattern. Next, the log latency was averaged over the 5 keypresses of each sequence. Those averaged log latencies were then anti-logged and multiplied by 5 to get the RT for each sequence. The graphed data points are those sequence RTs average over participants.

### Model fitting

The power component of Eq. (6a), (T-1)^-k^ ,would yield a divide by zero error for the pure case of (T-1), because on trial one, (T-1) would equal 0. To resolve that issue both here and in later described models that have T in the skill function, in the model fitting program we substituted (T–0.9999999999) for (T-1). This minor adjustment on the other model fits made no difference when using 1 or 0.999999999. Thus, for each equation where one was subtracted either from the trial number or cumulative sequence number, we used 0.999999999.

### Supplementary Information

Supplementary Information.

## Data Availability

All data and code (stimuli and analyses are available online (https://osf.io/j3uc8/). Further information and requests for all data and code should be directed to and will be fulfilled by the corresponding author, TCR (trickard@ucsd.edu).
