# Supplementary material for: Comparison of online, offline, and hybrid hypotheses of motor sequence learning using a quantitative model that incorporate reactive inhibition
Source: Sci Rep. 2024 Feb 26;14:4661. doi: 10.1038/s41598-024-52726-9 (PMC11269601; doi:10.1038/s41598-024-52726-9)
Supplement: Supplementary file 1 — Supplementary Information. [file 41598_2024_52726_MOESM1_ESM.docx]

**Supplementary**

For modeling and statistical tests, we removed the first sequence of each trial because that sequence had systematically longer RTs (Supp 1), as was done in Gupta and Rickard. Averaged across all trials, there is a significant difference for each group between the first and second sequence, t(41) = 10.2, p < .0001, d = 1.57; t(40) = 12.4, p < .0001, d = 1.94; t(41) = 9.69, p < .0001, d = 1.49; t(42) = 10.1, p < .0001, d = 1.53 for the S30, S10, M30 and M10 groups respectively. It is possible that since our data was collected online, this phenomenon is reflective of participants not paying attention at the beginning of each trial. However, the data from Buch et al., (2020), which was an in-person experiment, exhibit the same pattern or first sequence slowdown when plotting by sequence (Figure 1b). This was not noted in their original publication.
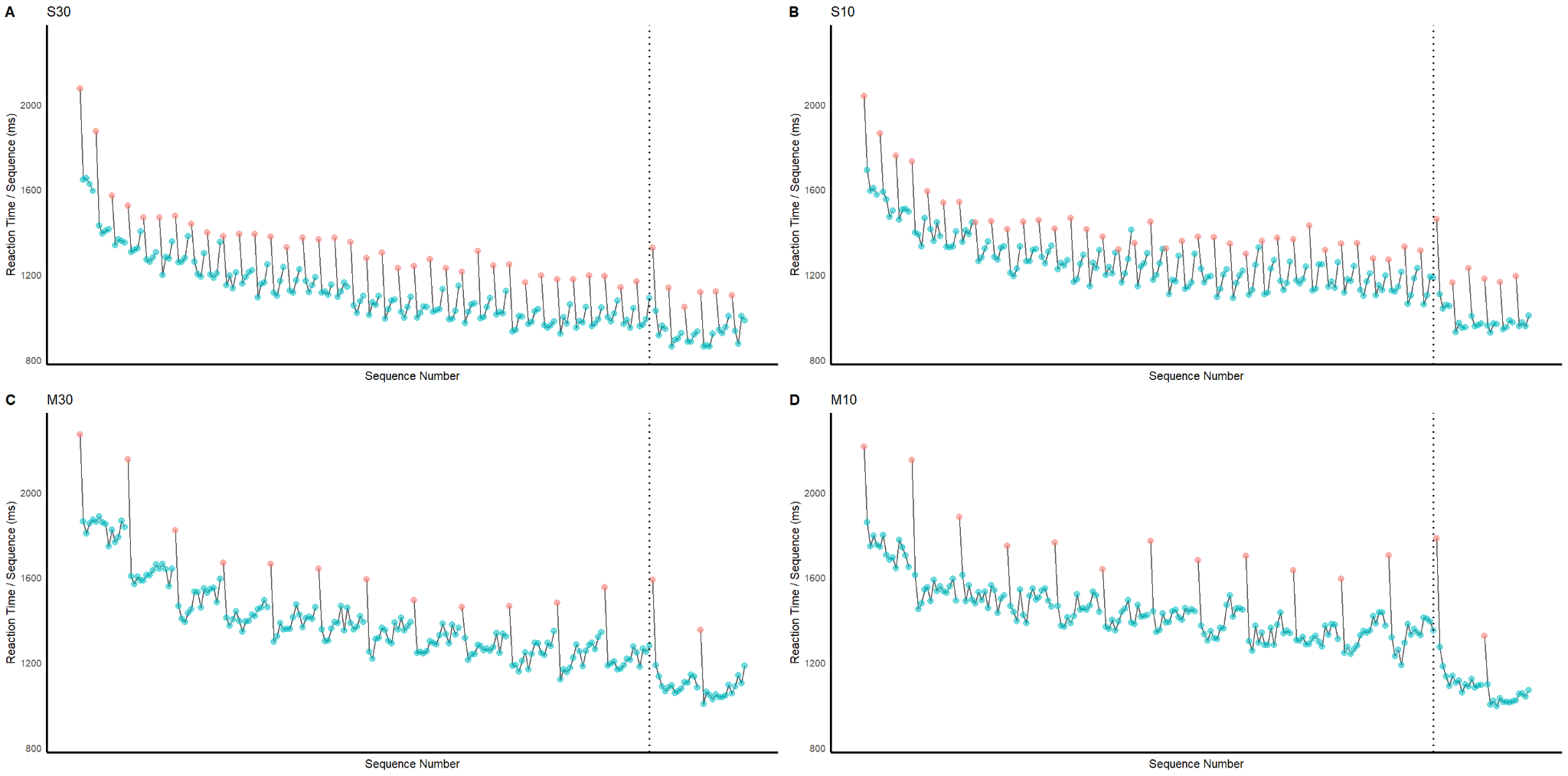


**Supp 1.** RTs for each sequence across trials, including the first sequence, marked in red. In every trial across groups, aside for the first couple, the first sequence is markedly slower than the rest. This systematicity warranted the removal of it because it is clear there is a third variable problem influencing the RTs of that sequence.

**Statistical Analysis of RI and Post-rest Gains**

The current method of controlling the number of sequences completed instead of controlling for the total practice time yielded analogous results to our previous study^2^, and in line with previous findings from Brawn et al., (2011) and Rickard et al., (2008). To confirm the presence of RI in the 15 sequence groups, we compared the second sequence to the 15th sequence averaged across all training trials. A one-tailed paired-samples t-test, averaged over all practice trials, yielded evidence of RI in the 30 s break group, t(41) = -3.83, p = .0002, d = -.42, but not the 10 s break group, t(42) = .33, p = .43, d = -.019. We believe this may be due to a consistently longer warmup time for this group. To confirm the presence of RI in the five sequence trial groups, we compared the second sequence to the last sequence. A one-tailed paired-samples t-test, averaged over all practice trials, yielded evidence of RI in the 30 s break group, t(41) = -2.94, p =.0027, d = -.32, and the 10 s break group t(40) = -6.35, p = <.0001, d = -.7.

We determined whether the current results replicate our previous findings regarding how the number of sequences completed and the break time affected the post-rest gain (Figure Supp 2). As in Gupta and Rickard (2022), we compared the RT means (sequence 2 onward) over the last two training trials (11 and 12) with the post-rest trials (13 and 14). A 2x2 mixed-factors Analysis of Variance (ANOVA) revealed a significant effect of break time on the post-rest gain, F(1, 164) = 9.08, p = .003, 𝜂^2^ = .05, as well as a significant effect of the number of sequences during the trial on the post-rest gain, F(1, 164) = 6.66, p = .012, 𝜂^2^ = .04 (Figure Supp 2). There was no significant interaction between the two factors, F(1, 164) = .321, p = .57, 𝜂^2^ = .002. These results replicated the same pattern observed in Gupta and Rickard.

**
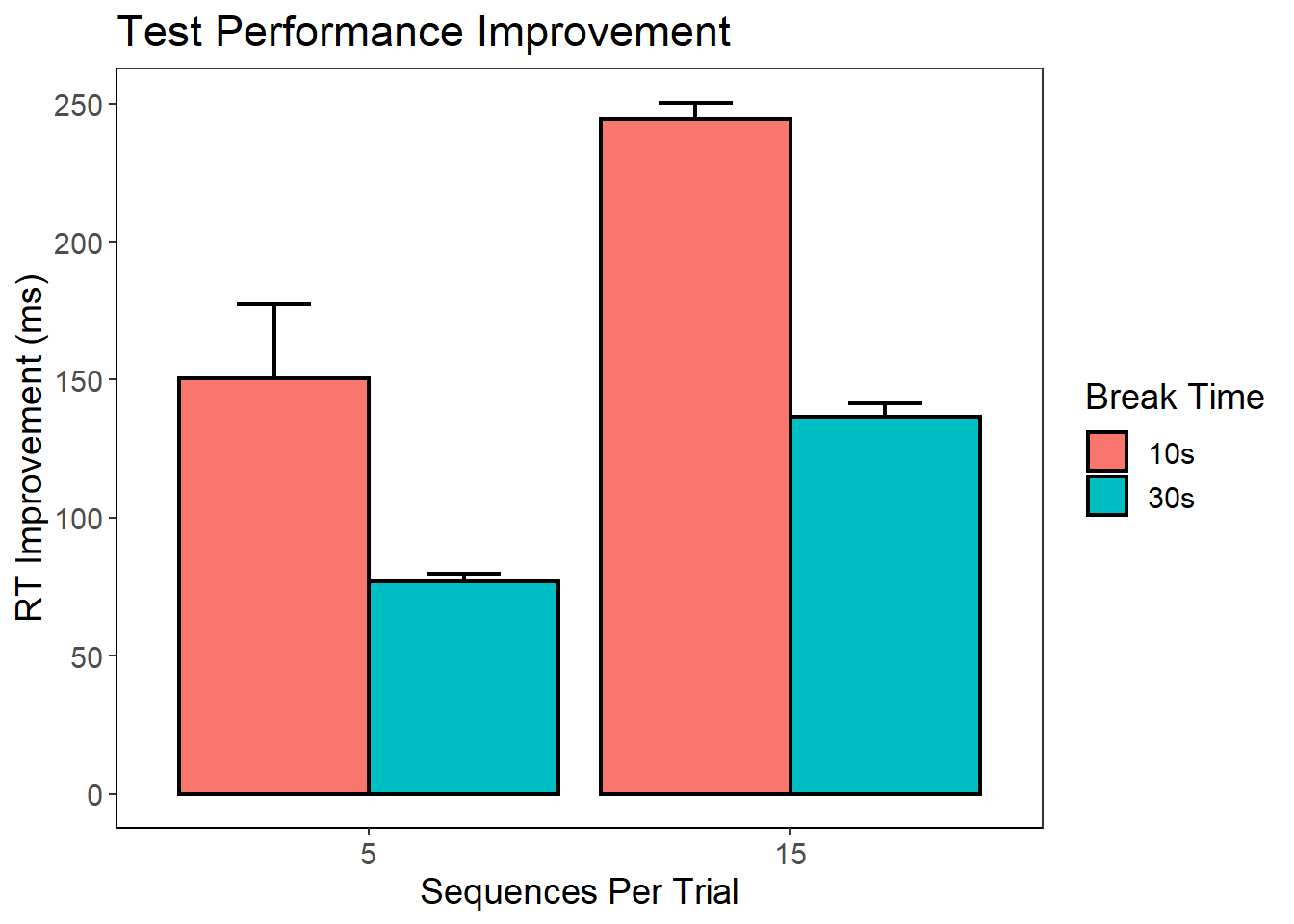
**

**Supp 2**. The bar chart shows the total amount of gain from the last 30 sequences of training compared to the 30 test sequences after rest. The y-axis is the average amount of RT gain. The x-axis indicates the number of sequences performed during a trial, whereas the color indicates the amount of break time. These results replicate our previous findings in Gupta and Rickard (2022) that both break time and the number of sequences performed affect the amount of gain.

**Parameter Necessity: Offline Model with RI**

We tested for improved fits with each added parameter by the BIC criterion, using the offline model version 2 as the reference. We started with the simplest case exponential RT gain parameter (*c*) only, with no RI (SM1), then added the within-trial RI parameter (*y*; SM2), then the cumulative RI parameter (*z*; SM3), and finally power RT gain parameter (*k*; SM4). As shown in Supp 3, for each group each added parameter reduced BIC.

| Group | SM1 | SM2 | SM3 | SM4 |
| --- | --- | --- | --- | --- |
| S30 | 1363.196 | 1303.667 | 1282.190 | 1241.242 |
| S10 | 1456.864 | 1416.645 | 1301.861 | 1279.505 |
| M30 | 1711.199 | 1632.545 | 1531.512 | 1514.275 |
| M10 | 1768.305 | 1748.233 | 1612.573 | 1583.348 |

**Supp 3**. *This table reflects the BIC values at each stage of the model development. Lower values indicate a better fit of the data for that model relative to other models.*

To obtain robust confidence intervals for the parameter estimates separately for each group, we performed bootstrapping, creating 999 sample datasets (Table 2). The pattern of parameter estimates provides insight regarding similarities and differences across groups.

| Model Type | Group | a | b | c | k | y | z | j | r^2^ |
| --- | --- | --- | --- | --- | --- | --- | --- | --- | --- |
| Online | S30 | 734.70 [.0051494, 816.80] | 940.01 [825.89, 1673.8] | .007042 [.0011389, .011652] | .15030 [.096666, .17675] | 28.52 [23.76,34.09 ] | 1.0328 [34.09, 1.742] |  | .78 |
|  | S10 | .2852 [0,770.1 ] | 1664 [913.1, 1749] | .001519 [.001163, .007562] | .08734 [.07120, .1386 ] | 32.25 [26.64, 37.75] | 4.286 [3.577, 5.011] |  | .69 |
|  | M30 | 891.7 [803.3, 942.3] | 1073 [985.2,1207 ] | .009453 [.006431,.01219 ] | .1020 [.07267, .1288] | 11.68 [10.18, 13.04] | 10.88 [8.635, 13.12] |  | .78 |
|  | M10 | .02784 [0, 783.9] | 1832 [1066, 1928] | .001782 [.001438,.005921] | .06570 [.05187, .09530] | 8.111 [6.322, 9.968] | 19.12 [16.83, 21.65] |  | .61 |
| Offline No RI | S30 | 863.3 [798.6, 904.4] | 556.9 [520.9, 606.2] | .04904 [.03694,.06071] | .01991 [.01558, .02406] |  |  |  | .66 |
|  | S10 | 0 [0,811.0] | 1430 [653.9, 1464] | .008461 [.007261, .02525] | .008306 [.005023, .01479] |  |  |  | .18 |
|  | M30 | 80.26 [0, 758.2] | 1516 [862.2, 1617] | .02884 [.02547, .06526] | .009336 [.007567, .01416] |  |  |  | .63 |
|  | M10 | 93.21[0,952.6] | 1519 [692.0, 1632] | .02630 [02183,.07940 ] | .005009 [.002930, .009278] |  |  |  | .094 |
| Offline | S30 | 825.7 [794.0, 854.7] | 571.2 [543.2, 596.8] | .05841 [.04845, .06876] | .01435 [.01161, .01722] | 23.62 [19.04, 28.50] | 1.390 [.6978, 1.979] |  | .80 |
|  | S10 | 54.47 [0, 800.8] | 1546 [796.1, 1620] | .005286 [.003678, .03276] | .1087 [.08642, .1707] | 27.23 [21.87, 32.95] | 4.431 [3.716, 5.207] |  | .66 |
|  | M30 | 431.8 [0, 900.7] | 1375 [960.6, 1805] | .01588 [.003483, .09875] | .2288 [.1686, .2950] | 6.853 [5.456, 8.227] | 10.45 [8.480, 12.46] |  | .81 |
|  | M10 | 3.227 [0,906.4 ] | 1685 [863.4, 1735] | .02034 [.01365, .1216] | .1084 [.06478, .1567] | 3.849 [1.966, 5.589] | 19.96 [17.37, 22.80] |  | .55 |
| HybridE | S30 | 745.3 [.1324,821.0 ] | 944.1 [847.9, 1665] | .02913 [.004932, .04626] | .1520 [.09923, .1778] | 26.57 [21.67, 32.06] | 1.066 [.3250, 1.697] |  | .78 |
|  | S10 | 81.67 [0, 776.6] | 1643 [924.3, 1753] | .006282 [.004606, .03105] | .08860 [.07074, .1367 ] | 30.56 [24.80, 36.44] | 4.290 [3.590,5.084] |  | .69 |
|  | M30 | 922.1 [861.7, 966.7] | 1170 [1100, 1252] | .1366 [.1043, .1711] | .1122 [.08739, .1362] | 8.592 [7.140, 10.05] | 11.23 [9.167, 13.34] |  | .80 |
|  | M10 | .01814 [0, 778.4] | 1871 [1143, 1963] | .02452 [.02, .07781] | .06658 [.05195, .09747] | 6.038 [4.136, 7.731] | 19.16 [16.69, 21.70] |  | .61 |
| HybridP | S30 | 716.3 [0,821.7 ] | 844.1 [730.6, 1562] | .005595 [.0007127, .01164] | .1873 [.1165, .2291] | 25.47 [19.99, 30.74] | 1.088 [.3808, 1.844] |  | .77 |
|  | S10 | 610.9 [0, 835.9] | 973.8 [752.2, 1606] | .003841 [.001005, .01017] | .1352 [.08664, .1821] | 28.99 [23.24, 35.22] | 4.531 [3.807, 5.374] |  | .66 |
|  | M30 | 689.6 [0, 892.56] | 1104 [915.5, 1797] | .002558 [.0002829, .00696] | .2610 [.1714, .3115] | 8.250 [6.592, 9.991] | 10.55 [8.591, 12.44] |  | .81 |
|  | M10 | 40.69 [0, 856.9] | 1647 [852.7, 1716] | .001494 [.0009690, .007239] | .1111 [.07706, .1626] | 5.636 [3.592, 7.778] | 19.82 [17.22, 22.68] |  | .55 |
| HybridJ | S30 | 820.6 [783.5, 850.5] | 598.4 [569.0, 627.8] | .01347 [.01110, .01600] | .02508 [.02045, .02982] | 23.88 [19.11, 28.60] | 1.297 [.6970, 1.935] | 4.097 [4.097, 4.097] | .80 |
|  | S10 | 598.9 [0, 805.4] | 1116 [786.3, 1702] | .003989 [.001314, .009086] | .1144 [.06449, .1409] | 31.91 [26.13, 37.65] | 4.414 [3.706, 5.177] | .1377 [0, 3.128] | .69 |
|  | M30 | 860.3 [713.9, 931.8] | 904.4 [800.3, 1083] | .007084 [.004475, .009520] | .06546 [.04074, .08926] | 7.997 [6.487, 9.570] | 9.990 [7.725, 11.87] | 13.42 [10.28, 14.88] | .82 |
|  | M10 | .1019 [0, 785.2] | 1647 [928.2, 1821] | .001850 [.001535, .005613] | .04025 [.02181, .07056] | 5.226 [3.239, 7.593] | 18.82 [16.48, 21.36] | 11.81 [4.623, 14.84] | .62 |

**Supp 4***.* Median parameter values are reported with 95% confidence intervals in brackets estimated from bootstrapping 999 samples. The number next to the letter is the amount of break time between trials. R^2^ is reported for model fits. Note that it is inappropriate to use R^2^ to compare non-linear least square models^4^.

**
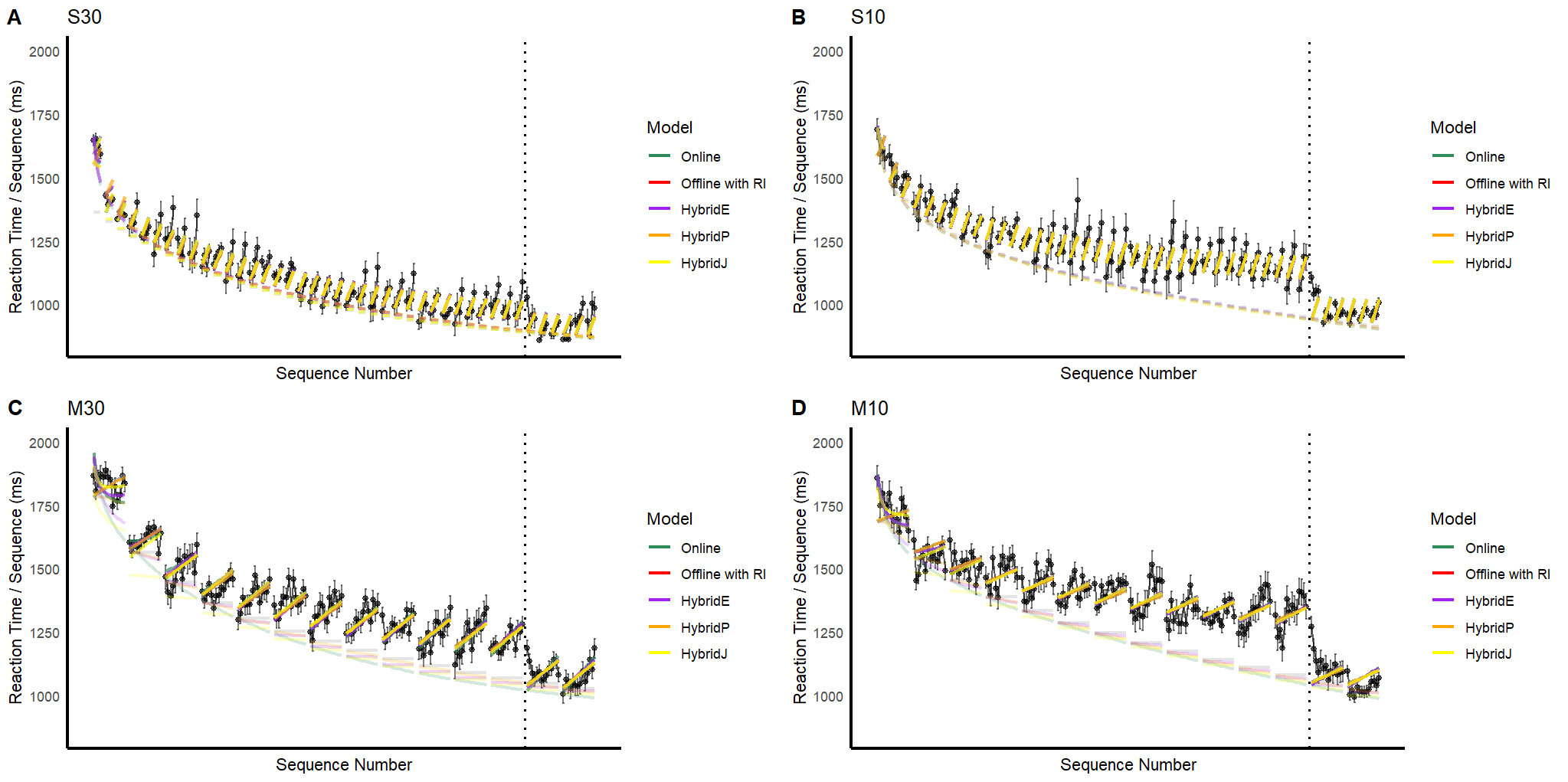
**

**Supp 5.** Each black dot is the RT of one completed sequence. Error bars are the standard error. This graph shows all model fits. The faint lines underneath are the estimated achieved skill.

*
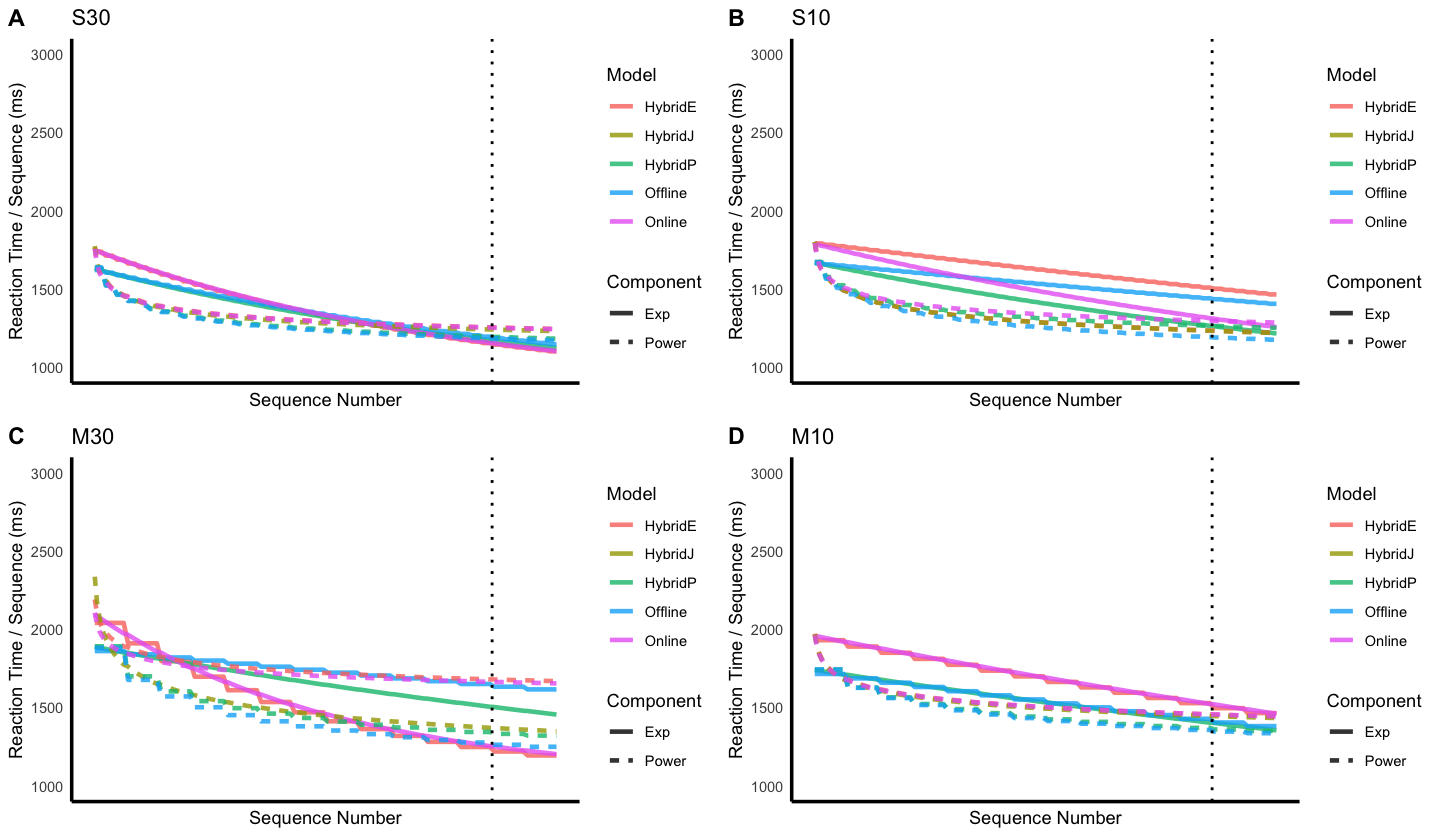
*

**Supp 6.** For each model, we plotted the exponential and power components plus the *a* and *b* parameters. For each model there appears to be a fast component that is typically the power learning rate and a slow component that is typically the exponential learning rate.

**Parameter Recovery**

To conduct parameter recovery, we used the bootstrapped parameter values that were used to calculate the confidence intervals of the models. Since we know the true parameter values, theoretically we should be able to fit the model again and recover the known parameter values. From those known parameter values, we simulated data for each model, for each group. We then injected uniform noise into the simulated dataset that was the plus or minus of the standard deviation of the entire RT of all sequences independently for each observed dataset. We ran this simulation 999 times to obtain estimates of the known parameter values. Finally, we correlated the recovered parameter values with the known real values.

*
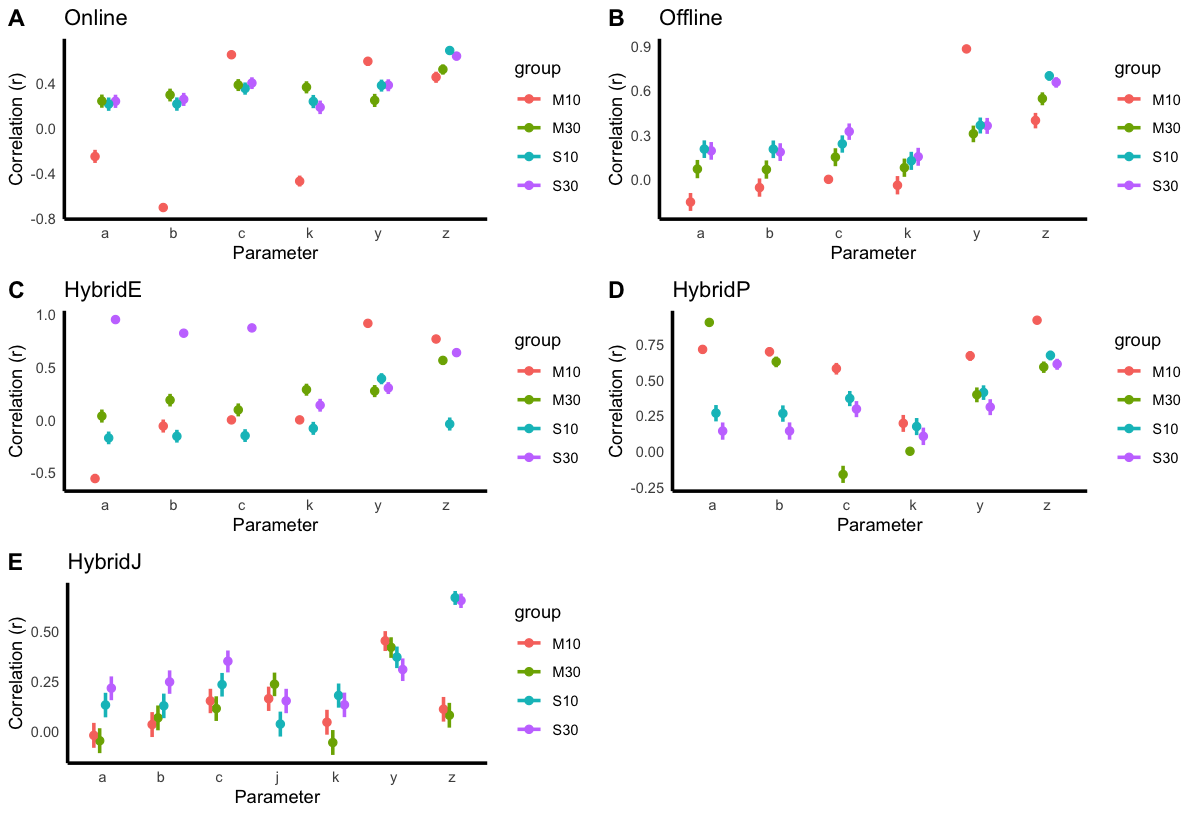
*

**Supp 7**. Each model underwent parameter recovery in which the recovered values were correlated with the known values. The lines indicate the confidence intervals for each correlation. For some parameters, in each recovery the same value was obtained (e.g. k = .1 for each recovered fit). In these cases we could not obtain sensible correlations. Thus, we filled those values 0s.


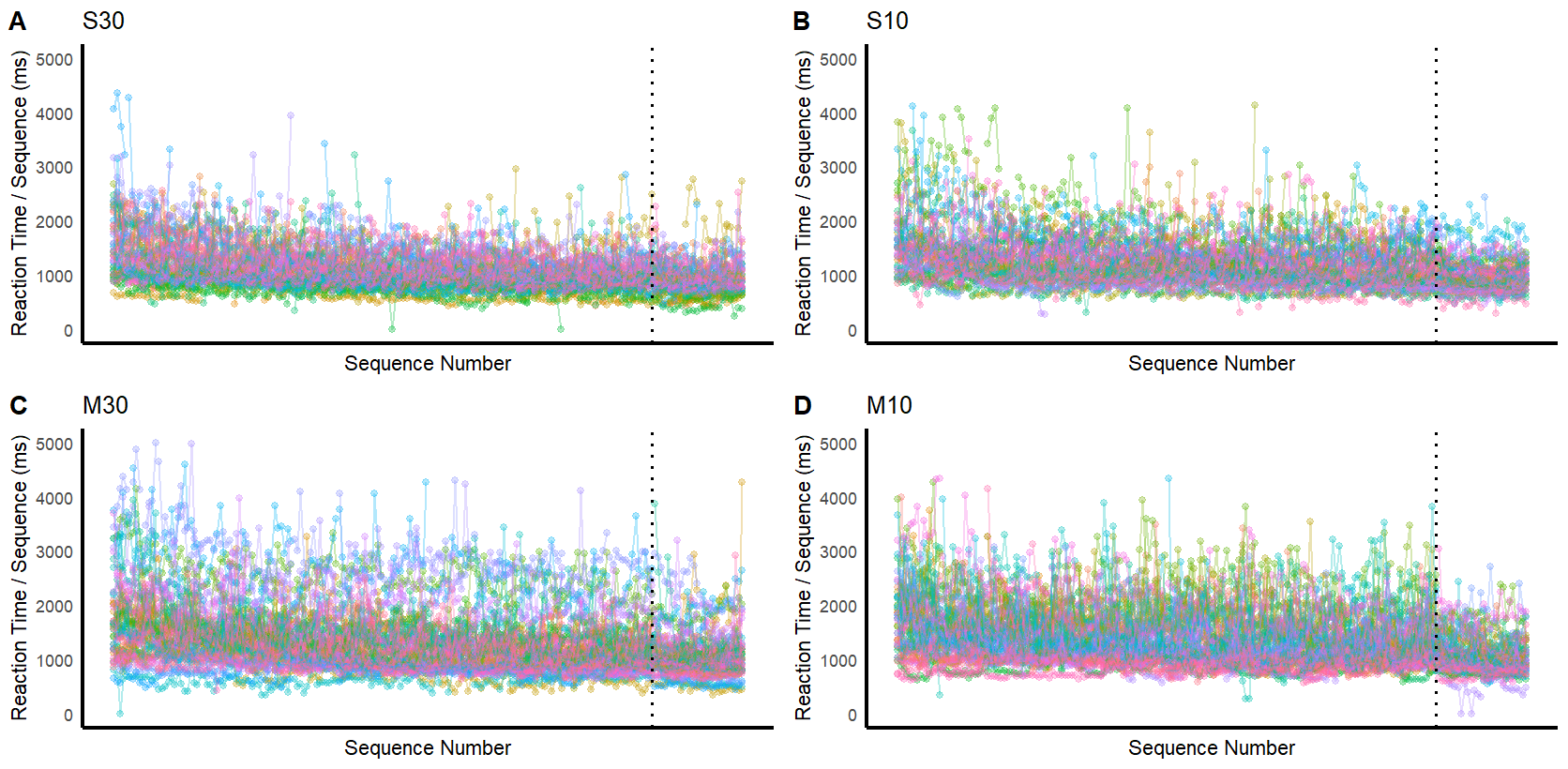


**Supp 8.** Individual participant sequence RTs for each group are plotted. Each participant has an individual color.

*
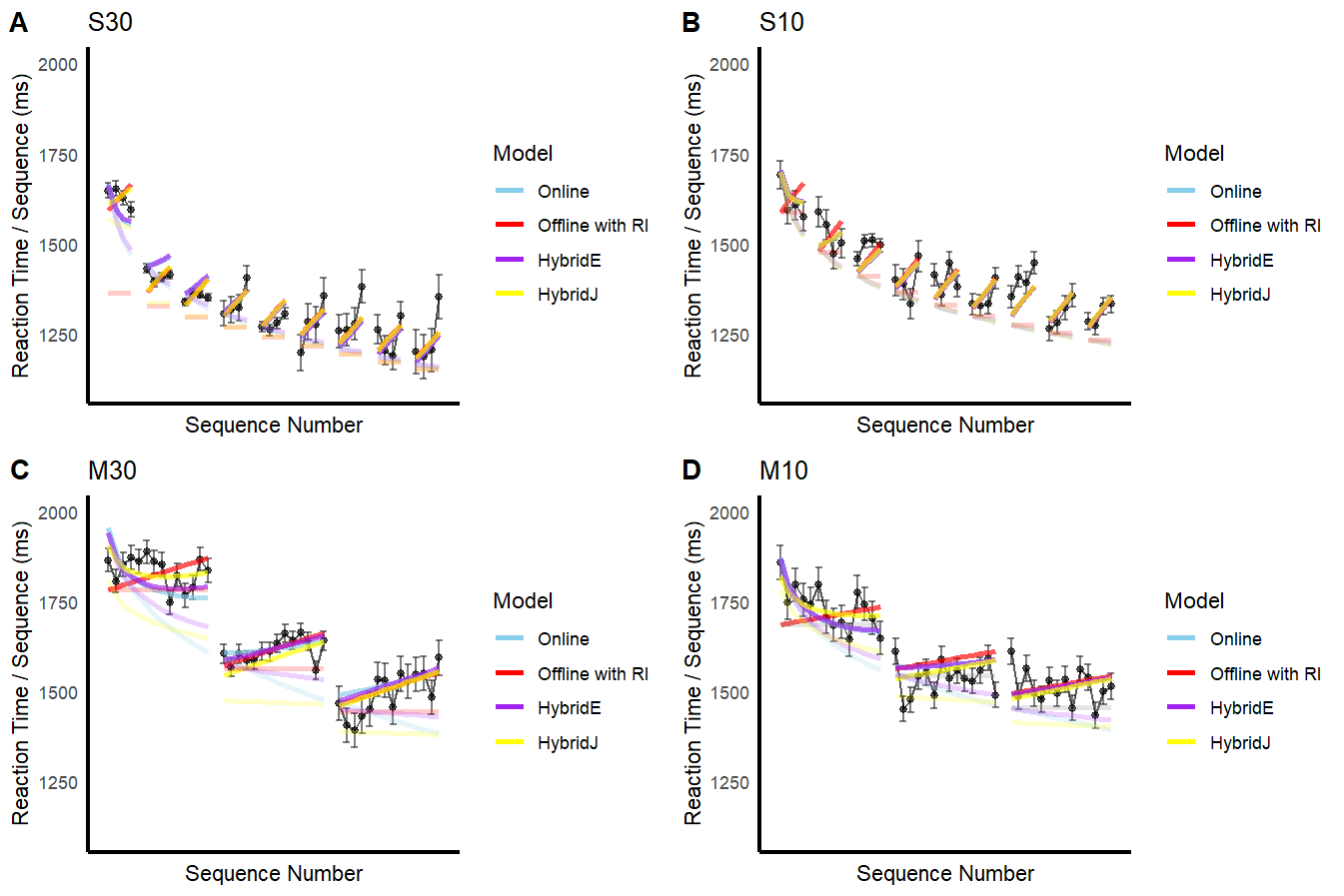
*

**Figure 9.** Each black dot is the RT of one completed sequence. Error bars are the standard error. This graph shows model fits over the first 3 (massed) or 9 (spaced) trials for online, offline with RI, HybridE, and HybridJ. The faint lines underneath are the estimated achieved skill. Much of the model differences occurred within these trials.

**References**

1. Gupta, M.W., Rickard, T.C. Dissipation of reactive inhibition is sufficient to explain post-rest improvements in motor sequence learning. *npj Sci. Learn.* **7**, 25 (2022). <https://doi.org/10.1038/s41539-022-00140-z>
2. Brawn, T. P., Fenn, K. M., Nusbaum, H. C. & Margoliash, D. Consolidating the effects of waking and sleep on motor-sequence learning. J. Neurosci. 30, 13977–13982 (2010).
3. Rickard, T. C., Cai, D. J., Rieth, C. A., Jones, J. & Ard, M. C. Sleep does not enhance motor sequence learning. J. Exp. Psychol. Learn Mem. Cogn. 34, 834–842 (2008).
4. Spiess A.N., Neumeyer N. An evaluation of R^2^ as an inadequate measure for nonlinear models in pharmacological and biochemical research: a Monte Carlo approach. BMC Pharmacol. 2010 Jun 7;10:6. doi: 10.1186/1471-2210-10-6. PMID: 20529254; PMCID: PMC2892436.
